# Retention of Urine from Stricture of the Urethra; Punctnre of the Bladder above the Pubis; “Extroducton” of the Bougie; Recovery

**Published:** 1849

**Authors:** Daniel Brainard

**Affiliations:** Professor of Surgery in Rush Medical College, Chicago


### ARTICLE V.

*Retention of Urine from Stricture of the Urethra; Punctnre of the Bladder above the Pubis; "Extroducton" of the Bougie; Recovery.* By DANIEL BRAINARD, M. D., Professor of Surgery in Rush Medical College, Chicago.

On the 6th of December, 1848 I was called to visit William Hickman, a stout, athletic, colored man, aged about 35 years, laboring under retention of urine from stricture. Eleven years previously he had gonorrhœa and stricture, which latter was neglected, although the surgeon in attendance gave full warning of the effects likely to result. From that time to the present he has been afflicted with the symptoms of stricture, which during the past year, have been severe, and for several weeks the retention has gradually become more perfect.

At present (Dec. 6th,) the bladder rises several inches above the pubis; there is great pain, and constant desire with inability to void the urine, except a few drops at a time. The urethra is hard, knotted, and irregular throughout its whole anterior part; the cellular tissues are much swollen, with a copious discharge of pus from the orifice. Abscesses had been, from time to time, formed along its course, and it was from one of these the pus came.

On attempting to introduce the catheter, it was found impossible to convey it more than three inches along the urethra before it entered a purulent cavity, behind which the canal could not be found.

Finding there was no prospect of relieving him in this way I ordered a saline purge, bled him to approaching syncope, and applied fomentations.

7th.—No relief. The bladder more distended, and some constitutional disturbance. Attempts to pass bougie repeated without success.

8th.—Symptoms urgent. The fundus of the bladder rises nearly to the umbilicus, and the urine is discharged guttatim. I now determined to puncture the bladder, above the pubis, with the long curved trochar. This was done with the aid of Prof. Evans and a number of pupils, although it was necessary to place the patient under the influence of chloroform which was done perfectly. The trochar was then introduced about an inch above the pubis, and the canula being carried to the bas fond of the bladder, the stylet, was withdrawn. After a copious discharge of urine, the orifice of the canula was closed with a small cork, which could be withdrawn for discharging the urine.

The unpleasant symptoms having, in this case, subsided in the course of twenty-four hours, the patient was extremely well satisfied with this new method of voiding urine, and would willingly have dispensed with further treatment. Injections were thrown into the urethra, and no further treatment used until the irritation of that canal had subsided. As soon as the state of the parts would permit, attempts were made to find the passage, and as often as the smallest sized bougie could be carried into any unexplored portion, it was dilated by using those of larger size from day to day.

The efforts to find the natural passage were continued by the use of every kind of bougie that promised success, for eight weeks; wax, gum elastic, catgut, and flexible bougies were repeatedly tried, and injections of water used without success. Near the posterior extremity of the spongy portion of the urethra was a knotty projection, beyond which the instrument would not pass.

Being upon the point of abandoning the case as incurable, the thought occurred that the prostatic and membranous portions of the urethra could be explored by means of an instrument carried into the bladder through the opening made by the puncture and still occupied by the canula. This being withdrawn, a small sized elastic bougie rendered firm by a good sized wire and bent to form a segment of a circle about a foot in diameter was passed into the bladder, and its point directed forwards. It was found very readily to enter the urethra, and passed with little difficulty, the obstacle which seemed insurmountable in front. By a little exertion, the point of the bougie was brought to within about two inches of the orifice of the urethra, and then seized with a pair of forceps, the wire was withdrawn, and the point of the bougie drawn out, the open end passing into and remaining in the bladder.

It was introduced in this manner Feb. 19th, 1849, and allowed to remain three days, when it was withdrawn and replaced by one of larger size, which passed without difficulty. Larger instruments were gradually used, till one above the medium size entered easily.

*March 4th.*---As much urine passes by the urethra as by the canula, and this was therefore withdrawn; after which the opening was closed, and urine passes more freely by the natural canal than has been done for a long time previously to the first operation.

He is now, March 31, 1849, pursuing his ordinary occupation, (shingle making,) and experiences little difficulty in voiding his urine. It is not to be expected, however, that any treatment could entirely restore the urethra to a healthy state but by an ocasional use of instruments, it is probable that the recurrence of so perfect a stricture may be prevented.

Several practical deductions may be made from this case. 1st. The puncture of the bladder above the pubis, if care be taken to prevent infiltration of urine, is a slight operation, and should not be deferred till extreme distention takes place. In further proof of this, we would refer to a case published not long since in the Buffalo Medical Journal, by our friend Dr. J. P. White, of that city. In that cuase a puncture of the kind served as a substitute for a urethra for a long time with but trifling inconvenience.

2d. This puncture may be made useful in catheterism. For this operation of passing a catheter forwards, which so far as we are aware, has not been done before. a friend has suggested the name of *Extroduction*. It may be performed with a properly curved instrument very readily, and if difficulties shuold occur, they may be obviated by passing the finger into the rectum. If the cases in which it is likely to be useful are rare, they are extremely urgent, and when they occur this operation, may prove a valuable means of relief.

